# Sharing is living: The role of habitat heterogeneity in the coexistence of closely related species

**DOI:** 10.1002/ece3.9930

**Published:** 2023-03-21

**Authors:** Fábio H. C. Sanches, Fernando R. De Grande, Tânia M. Costa, Rodrigo E. Barreto

**Affiliations:** ^1^ Institute of Marine Science Federal University of São Paulo (IMar/UNIFESP) Santos Brazil; ^2^ Postgraduate Program in Biological Sciences (Zoology), Biosciences Institute São Paulo State University (UNESP) Botucatu Brazil; ^3^ Biosciences Institute São Paulo State University (UNESP) – Coastal Campus São Vicente Brazil; ^4^ Aquaculture Center (CAUNESP) São Paulo State University (UNESP) Jaboticabal Brazil; ^5^ Department of Structural and Functional Biology, Biosciences Institute São Paulo State University (UNESP) Botucatu Brazil

**Keywords:** behavioral ecology, fiddler crab, habitat partition, heat stress, spatial distribution, sunlight shading, thermoregulation

## Abstract

In biologically diverse ecosystems, an essential process to support competing species to coexist is ecological differentiation. Habitat heterogeneity is, hence, important in establishing species abundance and richness, favoring the coexistence of species due to habitat partition. In this context, shading and species thermal tolerance can be good factors to elucidate the role of habitat heterogeneity in the habitat partition among closely related species. Herein, we study shading effects in microhabitat selection, behavior, and physiological limitation on two species of fiddler crabs (*Leptuca leptodactyla* and *Leptuca uruguayensis*). Indeed, shading conditions influenced fiddler crabs species proportion over time, with *L. leptodactyla* more associated with nonshaded/warmer areas while the *L. uruguayensis* to shaded/cooler ones. They also adjusted their behavior differently from each other to deal with thermal stress. Finally, we have demonstrated that these effects are related to species' physiological limitations. We conclude that biologically diverse ecosystems, such as intertidal regions from estuaries (e.g., mudflats and mangroves), support the coexistence between closely related species by reducing competition due to habitat partition.

## Graphical abstract

Our findings provide knowledge of the mechanisms behind the coexistence between estuarine sympatric species. We demonstrated that it is favored by the relationship between the diversification of microhabitats, such as shading degree, and by species’ intrinsic factors, such as thermal stress tolerance. Thus, habitat heterogeneity might favor species diversity in a general manner, due to reduced costs associated with habitat partition.

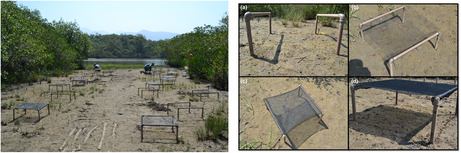

## 1 INTRODUCTION

Habitat heterogeneity and niche differentiation are fundamental for biological diversity, favoring the coexistence of closely related sympatric species (Brown et al., 2013; Castillo et al., 2021; Estevo et al., 2017; Tang & Zhou, 2011). Microhabitats' shading conditions and species' thermal tolerance may be important in this context. Shading may decrease the incidence of solar radiation and heat stress, affecting species differently (Kon et al., 2010; Munguia et al., 2017; Nobbs, 2003; Pardal‐Souza et al., 2016; Quinn et al., 1997). It may be advantageous for species that are not dependent on direct sunlight incidence or are susceptible to desiccation (Bennett et al., 2018; Principe et al., 2018; Sanborn et al., 2004), while it may be the opposite for primary producers or high‐temperature specialists (Koch et al., 2005; Lorda & Lafferty, 2012). The coexistence between closely related species in biologically diverse ecosystems, thus, could depend on microhabitats in habitat partitioning (as shading conditions) and species‐specific tolerance to environmental conditions (such as thermal stress).

Most terrestrial ecosystems are mosaic landscapes composed of patches of different levels of shading, including estuarine intertidal environments (Chou et al., 2019; Nobbs & Blamires, 2015). Estuaries are dynamic ecosystems with relatively heterogeneous biologically diverse microhabitats (Dame, 2008), which is fundamental for habitat partition (Nobbs & Blamires, 2015). Groups of species that are phylogenetically close may become shade‐patch specialists, so a competitive congener species can share the same ecosystem (e.g., mangrove forests, salt‐marshes fields) since they occur in different microhabitats (e.g., vegetated or nonvegetated) (Nobbs, 2003; Nobbs & Blamires, 2015). In this sense, restrictions in thermal tolerances may constrain some species to occupy the shaded areas in the vegetation or under the shade of natural or artificial structures (e.g., rocks, bridges, buildings), while other species tend to predominate in nonvegetated clearings at the edges of the forest or in sandbars (Checon & Costa, 2017, 2018; Lorda & Lafferty, 2012; Thurman et al., 2013). Therefore, these distributions of species can be linked to their intrinsic physiological limitations, influencing the decrease of interspecific competitive pressure in mixed‐species assemblies.

On a short‐term scale, species‐specific behavioral adjustments are also important to tolerate thermally adverse conditions among habitats with variations in shading degree. During the hottest parts of the day, some species of lizards need to retreat into cooler shelters to avoid overheating (Sinervo et al., 2010). By doing so, their foraging activities and reproductive behavior tend to be limited, which can negatively affect their fitness (Sinervo et al., 2010). The fiddler crab *Austruca mjoebergi* also altered its behavior in this context, remaining outside the burrows for longer periods foraging and searching for mates in shaded/cooler microhabitats than in nonshaded/warmer ones (Munguia et al., 2017). Similarly, females from the fiddler crab *Austruca lactea* adjust their rate of mate searching based on a size‐dependent temperature constraint (Takeshita, 2020). For a species of deposit‐feeding crab on a sandy shore (*Scopimera intermedia*), it has been shown that water uptake behaviors contribute to their thermoregulation, increasing their mating chances (Hui & Williams, 2021). Since thermal tolerance can positively influence feeding and mating opportunities, selection should favor individuals that remain for longer periods searching for mates, increasing reproductive success odds (Munguia et al., 2017).

Previous studies with intertidal benthic communities have demonstrated by a manipulative approach that artificial shading alters species distribution in different ecosystems, such as rocky shores (Pardal‐Souza et al., 2016) and mangroves (Kon et al., 2010; Nobbs, 2003). Although shaded‐patch dynamics is recognized as an important ecological factor in community structuring, most studies have addressed shading as a binary variable (i.e., presence/absence of shade; see Chou et al., 2019; Kon et al., 2010; Nobbs, 2003). Contrariwise, in the wild, shading is a graded factor. Some species may benefit by avoiding the extremes retreating to shaded areas (where primary producers are harmed) or keeping exposed to sunlight (where temperatures can become too high). The different shading degrees may therefore represent specific microhabitats, and studies that investigate these gradients are required to better understand the distribution and habitat sharing of organisms.

Given the context, fiddler crabs are good models of organisms for understanding associated tradeoffs in other intertidal ectothermic organisms. For example, the fiddler crabs *Leptuca leptodactyla* and *Leptuca uruguayensis* are closely related species that share sandy sediment banks along with mangrove forests from Southwestern Atlantic (Fogo et al., 2019; Machado et al., 2013; Thurman et al., 2013). While *L. leptodactyla* tends to predominate in nonvegetated clearings at the edges of the forest or in sandbars, *L. uruguayensis* tends to occupy the shaded areas from the interior of the vegetation (Checon & Costa, 2017, 2018; Thurman et al., 2013) or under the shade of artificial structures (e.g., bridges, buildings; personal observation). Both species can occur in mixed assemblies for which it has been reported that males compete extensively for space and resources (Fogo et al., 2019). Therefore, it is expected that the degree of shading might play a role in both species' distribution and that their behavior and physiological aspects are distinct when exposed to thermal stress.

Herein, our study aims to evaluate whether habitat partition between intertidal closely related species is influenced by the diversification of microhabitats characteristics (i.e., habitat heterogeneity), such as sunlight shading, as well as species tolerance to environmental conditions, such as thermal stress. As a model, we used the sympatric congener species of fiddler crabs *L. leptodactyla* and *L. uruguayensis*. Specifically, we tested: (i) the shading gradient effects in microhabitat selection; (ii) the temperature effects on species behavior; and (iii) the divergence in physiological limitation between species.

## 2 METHODS

### 2.1 Species and study site

Herein, it was studied a fiddler crab assembly mainly composed of the two model species: *L. leptodactyla* and *L. uruguayensis*. The first one is a tropical‐subtropical species that occurs from the state of Florida—the United States of America—to the state of Santa Catarina—Brazil; yet the second is a subtropical‐temperate species that occurs from the state of Rio de Janeiro—Brazil—to the city of Buenos Aires—Argentina (Thurman et al., 2013).

**FIGURE A1:**
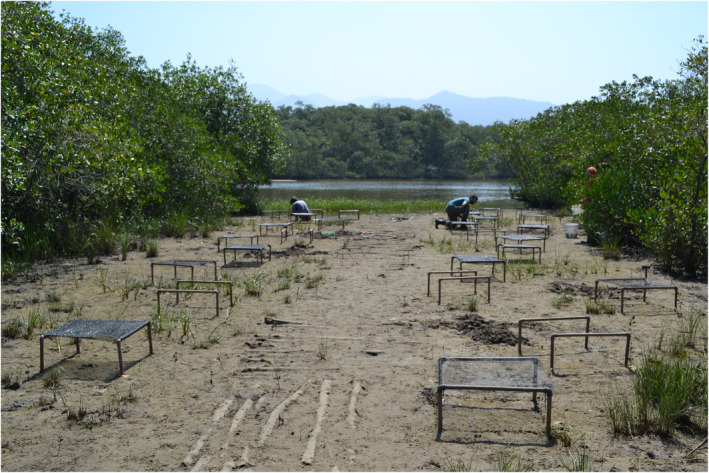
Overview of the study site where we observed the shading effect on species microhabitat selection.

The study site was an estuarine region under a semi‐diurnal tide system, located at Una do Prelado River, south coast of the state of São Paulo—Brazil. This region is a conservation unit named Ecologic Station Juréia—Itatins (24°26′18.11″ S, 47°04′20.41″ W). The area was a muddy‐sand bank closer to the mouth of the river, with a rectangular open clearing (about 30 m length × 10 m width) surrounded by mangrove vegetation (Appendix 1: Figure A1). The site was completely flooded by water during the spring high tides (every 6 h). Nevertheless, the site was exposed to the sun during the neap tides and spring low tides. Overall crab density (mean ± SD) was 106.09 ± 16.43 crab per m^2^ (*L. leptodactyla*: 94.14 ± 17.98; *L. uruguayensis*: 11.95 ± 12.17), sampled with five quadrants of 0.25 m^2^, in seven different periods from August 2014 to June 2015 (*n* = 35 quadrants).

### 2.2 Shading effect on species microhabitat selection

The first experiment tested how shading gradient affects species microhabitat selection (performed from August 2014 to September 2015). The experiment consisted of controlling the luminous intensity in four different levels of shading to observe changes in the proportion between *L. leptodactyla* and *L. uruguayensis* distribution throughout time (adapted from Nobbs, 2003). For this purpose, it was provided artificial shading structures covered with four different levels of shading: 0% of light filter (nonshaded); 20% light filter; 50% light filter; and 80% light filter. The crabs' abundance was sampled inside each structure before the study started, followed by 1, 2, 3, 5, 7, 9, and 13 months after the experiment had started. Since the estuarine areas are dynamic, some structures have suffered some damage, so the sample size was 6–7 replicates per shading treatment on each time series period (there were three damaged structures: one for unshaded, one for 50% light filter, and one for 80% light filter treatments).

**FIGURE 1:**
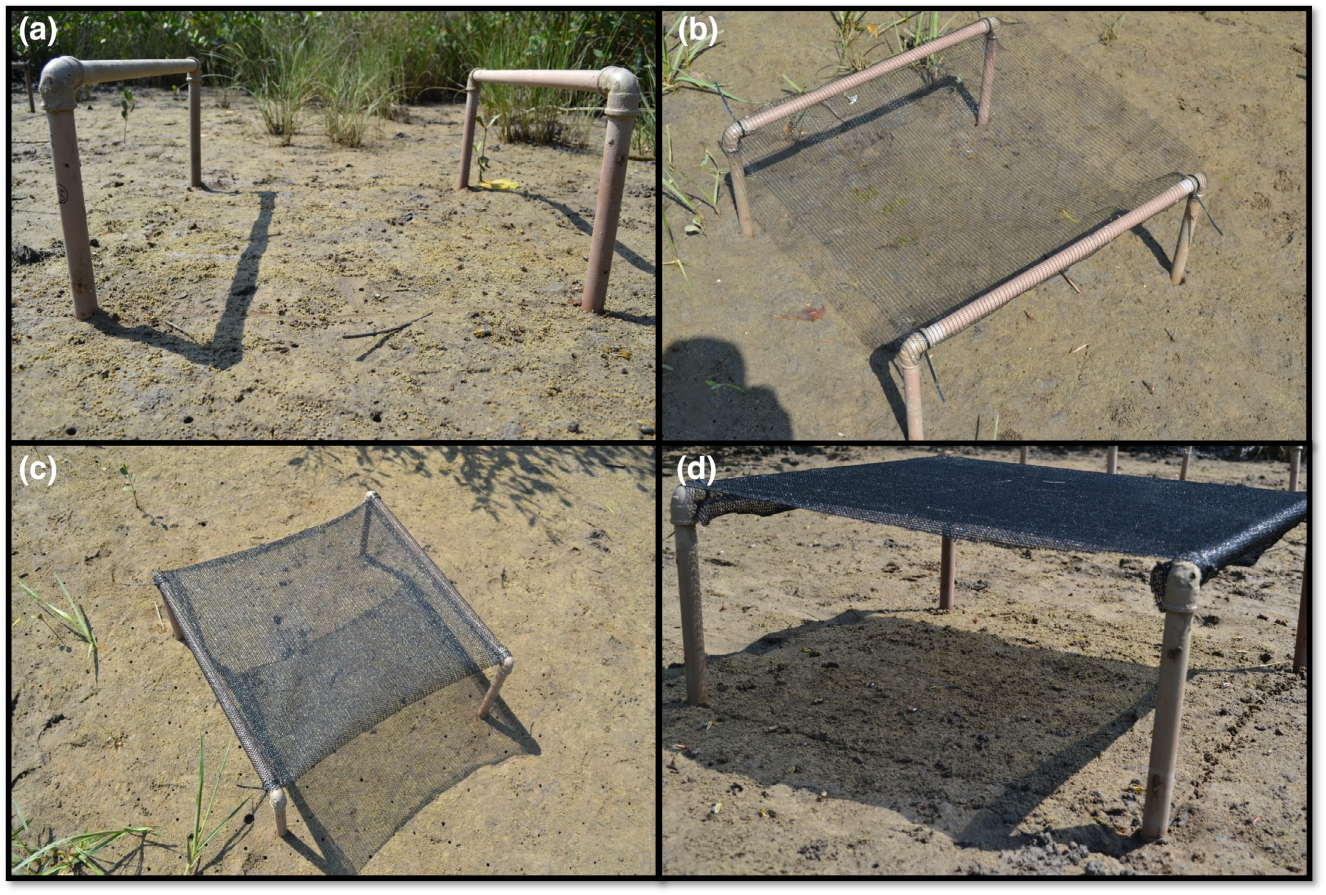
Experimental shading structures covered by four different levels of shading: (a) 0% of light absorption (nonshaded control group); (b) 20% of light absorption; (c) 50% of light absorption; and (d) 80% of light absorption.

The structures were made with four polyvinyl chloride pipes with a 3.2 cm diameter, buried by 35 cm, forming a square of 60 cm × 60 cm. On the top of these pipes, two parallel pipes were attached by pipe knees, and each polyester shade cloth with different mesh sizes was tied with nylon fishing lines to the pipes, with the cover suspended 25 cm from the soil (Figure 1). The crabs' number and species were sampled by observation (waiting period of 5 min, 2 m distant from the shading structures) during spring low tides (always between 10 and 13 h), to avoid manipulation interferences in the population throughout the experiment (Nobbs & McGuinness, 1999). Only male crabs from our study models were counted to avoid female misidentification (similar shape and colors from both species).

### 2.3 Shading effect on sediment abiotic factors

Sediment surface temperature and luminous intensity were measured to confirm the effectiveness of the shading treatment. They were sampled at three different periods from each shading structure (between 10 and 13 h), during spring low tides of random sunny days (from October to December 2016). Soil temperatures were measured in the center of each shading structure with thermosensors wires of a digital thermometer (0.1°C; LutronTM, TM‐947SD). The luminous intensity was also measured in the center of the structures close to the sediment, with a digital lux meter (0–99,999 lux; Testo 540). The sample size was 1–2 measurements per shading treatment per day (sample size ranging from 5 to 14 random measurements in each shading structure per time).

Sediment samples were collected from the structures to check sediment surface organic matter content from each shading treatment. They were sampled at five different times (between 10 and 13 h), during spring low tides (from September 2014 to June 2015). Three random sediment samples (composite samples) were collected with a 3.2 cm diameter core (5 mm depth) under all structures of each experimental treatment. After each sediment sample collection, they were bagged, labeled, and frozen for laboratory analysis. The samples were dried in the laboratory at 60°C for 72 h in an air circulation oven, weighted ~10 g from the dry material, incinerated at 550°C for 5 h in a muffle, and reweighted to obtain the organic matter content (%) by the differences between samples (Natálio et al., 2017). The sample size was one composite sample from each of the seven structures from the four experimental groups, during five different periods. All samples were collected after observing the crabs' distribution in the shading experiment, without disturbing their burrows.

### 2.4 Behavioral responses concerning the temperature

Male crabs' behaviors were observed from both species (between October and December of 2016), in random nonshaded sites, and during spring low tide on three different days (from 3 to 5 samples per day, from 10 to 13 h). It was considered the sediment surface temperature, crabs' further distance traveled from their burrow entrance, as well as the time spent outside their burrows. Therefore, we compared *L. leptodactyla* versus *L. uruguayensis* behavior concerning the soil temperature. The sample size was 26 for both species.

Behaviors were recorded with a video camera (Sony DCR SR68) in a tripod over the sediment surface (2–3 individuals of each species were observed per video of ~40 min). The sediment surface temperatures were measured at the start and end of, respectively, filming. Crabs' traveled distances were measured with a known scale on each record. The images were evaluated using ImageJ (National Institutes of Health) when crabs were in the further position from their burrow. Concerning the time spent outside the burrows, it was analyzed random crabs, starting when they emerged until they retreated to their burrows. Animals that retreated into their burrows (due to the influence of competing males) or suffered influence on the distance reached (due to mating‐search females) were discarded from the analyses.

### 2.5 Species physiological limitation

#### 2.5.1 Water loss

Male crabs from both species were submitted to three different air temperatures similar to the field (25°C, 30°C, and 35°C), and their water loss was measured throughout time: at the start of the experiment, as well as 5, 15, 30, 60, and 120 min after the experiment start (based on Levinton et al., 2015). Mortality from each species was also counted for each treatment at the end of the experiment. The sample size was 6–12 crabs at each temperature in the six time periods.

Crabs of similar size length (*L. leptodactyla* mean CW ± SD of 9.06 ± 0.436 mm; and *L. uruguayensis* mean CW ± SD of 9.16 ± 0.702 mm; *t*‐test, *t*_56_ = −0.6514, *p* = .518) were manually sampled and kept in species‐specific buckets (diameter: 35 cm, height: 30 cm, 10 cm sediment layer), with crabs' density similar to the field (106.09 ± 16.43). Crabs from each stock population were placed in a glass tank (40 × 24 × 23 cm) for 48–72 h containing 1 cm deep brackish water for them to release their feces and to stay similarly hydrated. Next, a dry glass tank was covered with paper towels where crabs were placed for 1 h to ensure dryness, with laboratory temperature ~25°C. Crabs were weighted at the experiment start (digital balance of 0.0001 g precision) and placed inside an air circulation oven at the three different temperatures. They were reweighted in each of the five periods after the start of the experiment.

#### 2.5.2 Thermal limits

The last experiment tested males' crab critical thermal maximum (CT_max_) to evaluate whether *L. uruguayensis* are more sensitive to heat than *L. leptodactyla*. The experiment consisted of raising the water temperature from 25°C to 50°C till the crabs' motor coordination is lost (De Grande et al., 2021). The CT_max_ was calculated as the average temperature at which individuals were not able to recover their position (Allen et al., 2012). The sample size was 15 for each species.

Similar‐sized crabs from both species (*L. leptodactyla* mean CW ± SD of 7.71 ± 0.61 mm; and *L. uruguayensis* mean CW ± SD of 7.90 ± 0.48 mm; *t*‐test, *t*_28_ = −0.9172, *p* = .3971) were sampled in the same field site of the previous experiments and acclimated individually in glass tanks (10 × 10 × 4 cm) filled with 1 cm deep brackish water placed for 48 h in a controlled germination chamber at 25°C. Later, each crab was relocated to circular glass containers of 40 mm diameter, filled with 5 mL of brackish water (to cover the fiddler crabs' ventral portion), and placed in a water bath. This ensured that the body was similar to the water temperature (Darnell et al., 2015; Darnell & Darnell, 2018). The temperature was raised at a rate of 0.3°C min^−1^, from 25°C to 50°C, till they lost their motor coordination capacity. When temperatures were above 40°C, crabs were rapidly removed and placed on their back inside their container every minute. The ones that were able to recover position back were instantly returned to the water, while those that could not recover in 15 s range were removed from the experiment to return to a normal condition, being released in the environment after the experiment had finished (Allen et al., 2012).

### 2.6 Data analysis

Most of the data were analyzed by generalized linear mixed models (GLMM). There were two exceptions: the mortality data from the *Water loss* experiment, which was analyzed by Chi‐squared test for given probabilities for the temperature of 30°C (since no crabs died at 25°C and all crabs died at 35°C); and the data from the *Thermal limit* experiment, in which a Student's *t*‐test was used to compare the CT_max_ between *L. leptodactyla* and *L. uruguayensis*.

For the GLMM analysis, a subset of different models was chosen by using the Akaike information criterion (Burnham & Anderson, 1998; see model selection in Appendix 2). For the *Shading effect on species microhabitat selection* experiment, the bound proportion between *L. leptodactyla* and *L. uruguayensis* (i.e., cbind function in R) was the response variable, with a binomial family and a logit link function. As the experiment was performed in a nonvegetated sandbar mainly composed of *L. leptodactyla*, we considered the presence of this species as a “success,” while the presence of *L. uruguayensis* as a “failure.” It was selected as a random slope model (random factor: structure IDǀtime; i.e., repeated measure), with shading level as fixed factors (0%, 20%, 50%, 80%), and time as a continuous variable. Regarding the *Shading effect on sediment abiotic factors*, soil temperature and luminous intensity were the response variables, with a Gamma family and log link function. The shading level was the fixed factor and time was the random factor (1ǀtime). This was because we measured randomly under the structures and on different days only as replicates (with no hypothesis on time series). In both cases, it was used Fisher LSD as a post hoc test. Furthermore, the percentage of organic matter content in the sediment was also the response variable, with a Gamma family and a log link function. In this case, it was selected a random intercept model, with the random factors structure identity (1ǀID) and time (1ǀtime), while the fixed factor was shading level. For the *Behavioral responses concerning the temperature* experiment, the time crabs spent outside their burrows and the further distance traveled were the response variables (both Gamma families with log link function). It was selected a polynomial model to the first one, with crab identity as a random factor (1ǀID), the sediment surface temperature as a covariate (polynomial), and the species as a fixed factor, while the second one was a similar‐structured nonpolynomial random intercept model. Finally, for the *Water loss* experiment, the percentage of body water was the response variable (Beta family with logit link function). It was selected a random slope model (random factor: crab IDǀtime), with the fixed factors temperature, species, and time.

All data were checked for the assumptions through visual inspection (standardized residuals normally distributed and the scatterplot of predicted values against residuals with a shotgun pattern). Analysis was performed in R software version 3.6.3 using the package “lme4” (Bates et al., 2015), GLMMadaptive (Rizopoulos, 2022), and figures with “ggplot2” (Wickham, 2016). Statistical significance was considered when *p* < .05 in all analyses.

### 2.7 Ethical note

This study had a license to collect, maintain, and perform experiments with invertebrates in the laboratory from the Authorization and Information System in Biodiversity (SISBio; protocol number 42907). It also had the authorization to conduct field experiments in Conservation Unities from the Technical Scientific Committee (COTEC; protocol number 260108—002.036/2014).

## 3 RESULTS

### 3.1 Shading effect on species microhabitat selection

**FIGURE 2:**
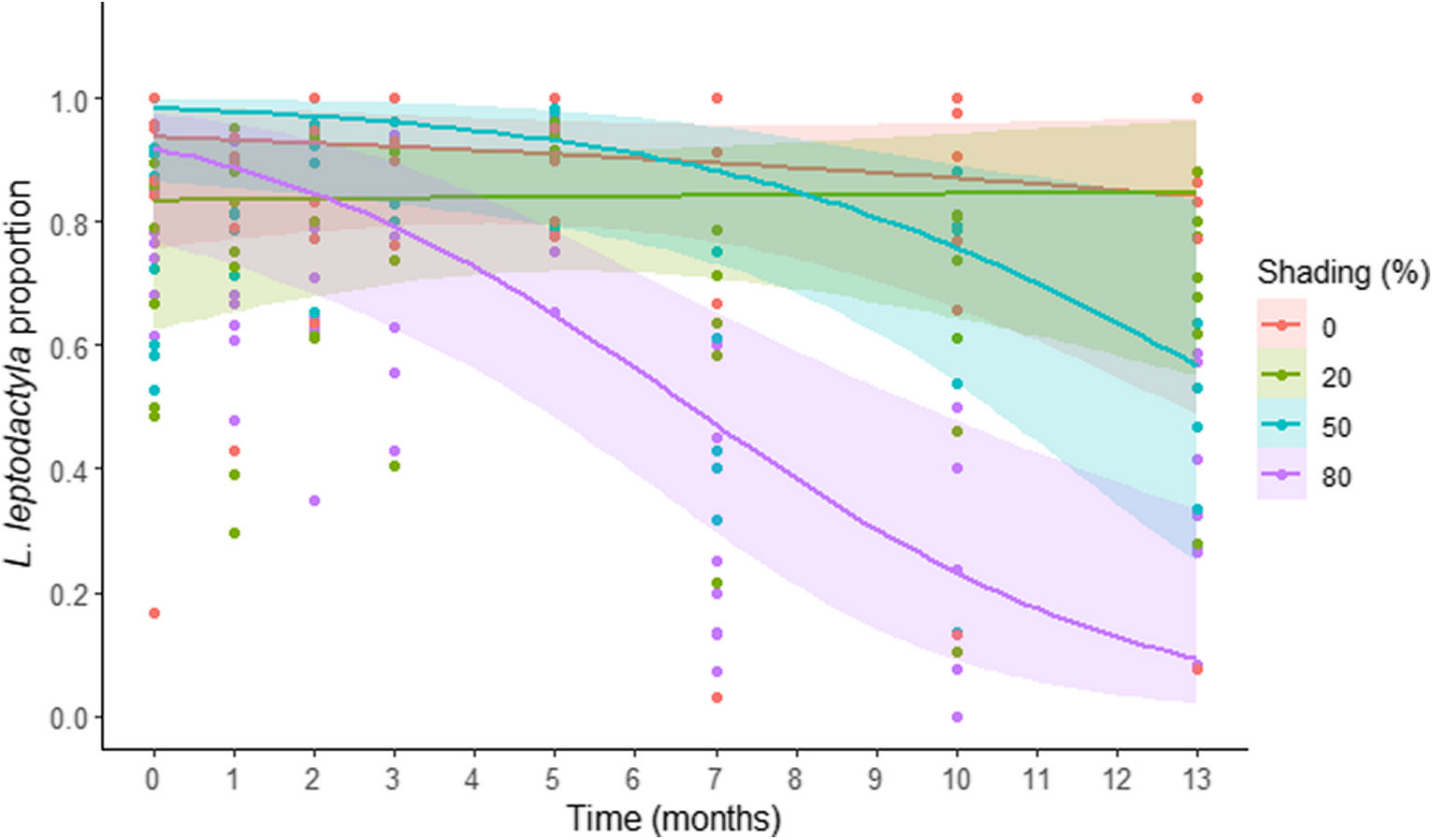
*Leptuca leptodactyla* proportion throughout time concerning the shading gradient treatments. Dots are the proportion from each experimental structure identity, while lines are the binomial logistic regression, and shaded areas represent the confidence interval of 95%. *Note*: All percentage values in the text are related to the mean ± SE from the *L. leptodactyla* proportion under each experimental structure. Nevertheless, data were analyzed with a GLMM with a binomial distribution. Thus, the lines drawn herein had different values from the mean since they are related to the binomial logistic regression.

**FIGURE A2:**
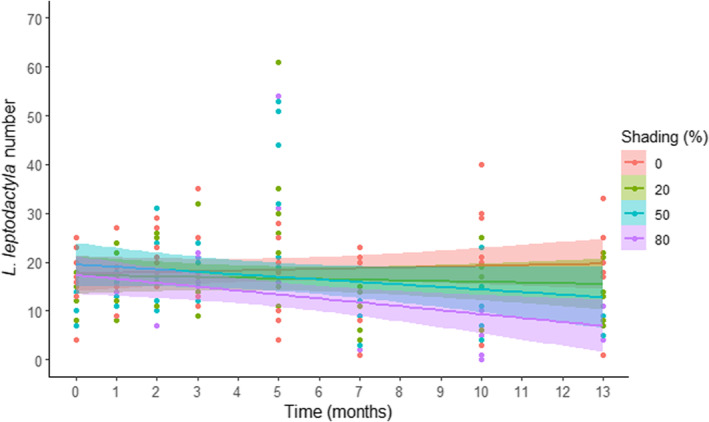
*Leptuca leptodactyla* raw number throughout time concerning the shading gradient treatments. Dots are the number of crabs from each experimental structure identity, while lines and shaded areas represent the linear regression ± SE.

**FIGURE A3:**
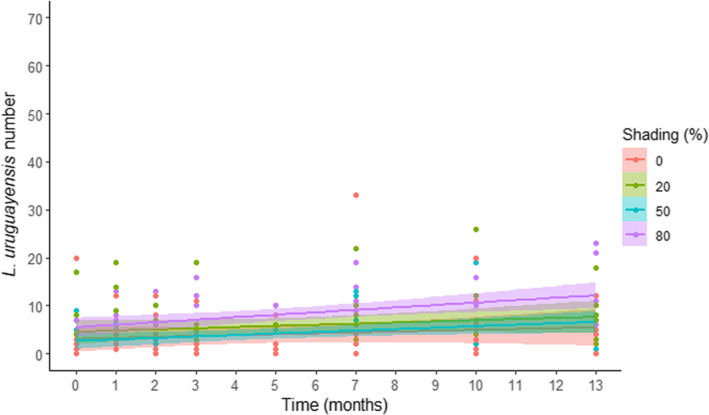
*Leptuca uruguayensis* raw number throughout time concerning the shading gradient treatments. Dots are the number of crabs from each experimental structure identity, while lines and shaded areas represent the linear regression ± SE.

Concerning the shading effect on species microhabitat selection (see Appendix 1: Figures A2 and A3 for crabs' number raw data; Appendix 2: Table A1 for model selection), there was an interaction between the fixed factor shading and the covariate time. *Leptuca leptodactyla* in relation to *L. uruguayensis* decreased from 78 ± 5% (mean ± SE) to 36 ± 7% after 13 months in the 80% of shading level treatment, while for the shading of 50% (from 73 ± 6% to 61 ± 8%) and 20% (from 72 ± 6% to 68 ± 7%), it was similar to the control group (from 83 ± 11% to 76 ± 14%; Figure 2; Table 1).

**TABLE 1:** Generalized linear mixed models testing the effect of shading level (0%, 20%, 50%, or 80%) and time on *Leptuca leptodactyla* proportion.

*Leptuca leptodactyla* proportion				
Parameters	Estimate	SE	*Z*‐value	*p*‐Value
*Fixed effects*				
(Intercept)	2.4	.31	7.72	**<.001**
Time	−0.06	.03	−1.80	.071
Shade 20%	−0.81	.42	−1.92	.055
Shade 50%	−0.34	.43	−0.79	.429
**Shade 80%**	**−1.13**	**.42**	**−2.72**	**.007**
Time*Shade 20%	0.01	.04	0.23	.817
Time*Shade 50%	−0.03	.04	−0.66	.511
Time*Shade 80%	**−0.10**	**.04**	**−2.30**	**.021**

Parameters		Variance	SD	Corr
*Random effects*				
ID	(Intercept)	0.465	0.682	
	Time	0.003	0.055	.18
Observations	217			
*N*_ID_	28			

*Note*: The selected model structure was: time + shade + time:shade + (time|id). The bold font indicates statistical significance (*p* < .05).

Abbreviations: Corr, correlation coefficient; ID, experimental structure identity; *N*, sample size; SD, standard deviation; SE, standard error.

### 3.2 Shading effect on sediment abiotic factors

It was observed a graded effect of all four degrees of shading in both sediment surface temperature and luminous intensity, with the most shaded areas with lower temperatures and luminous intensity, while higher values for both variables were observed in the nonshaded treatments (Table 2).

**TABLE 2:** Mean ± SD of soil temperature and luminous intensity from the four shading structures treatments (0%, 20%, 50%, 80%).

Treatment	*N*	Soil temperature (°C)	Luminous intensity (lux)
0%	39	35.23 ± 4.01 a	91,045.64 ± 20,937.48 a
20%	30	32.07 ± 4.12 b	73,552.43 ± 22,939.58 b
50%	35	29.12 ± 3.04 c	40,107.29 ± 13,718.91 c
80%	42	27.26 ± 2.21 d	16,273.49 ± 5107.29 d

*Note*: The selected model structure was: shade + (1|time). The different letter means the statistical difference between treatments for the same variable (Fisher's LSD test; *p* < .05).

**FIGURE 3:**
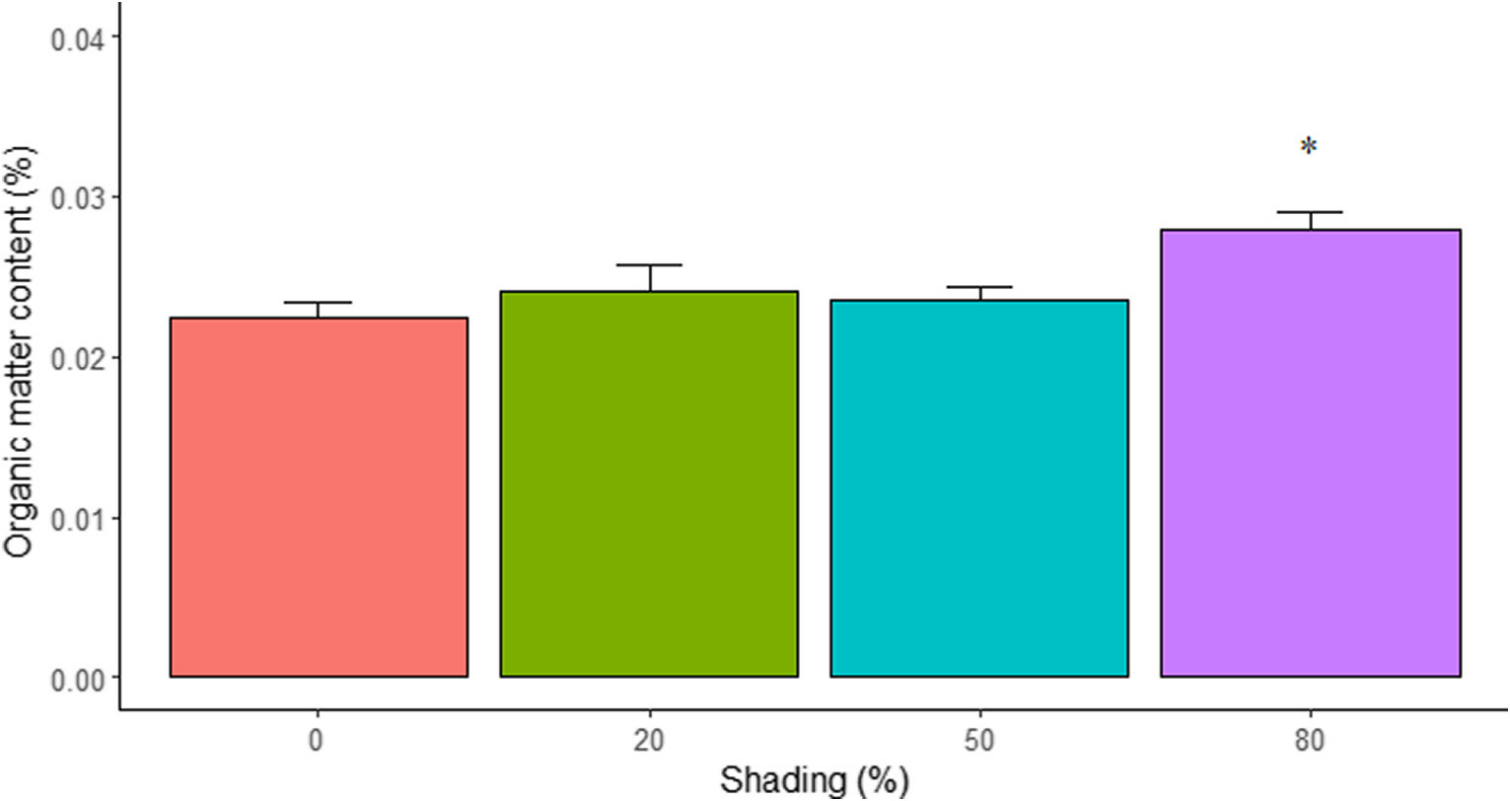
Mean ± SE for the sediment organic matter content under the shading treatments. The asterisk denotes statistical differences in comparison with the control group (*p* < .05).

Regarding the sediment organic matter content under the shading treatments (see Appendix 2: Table A2 for model selection), there was an effect from the fixed factor shade, with higher values of sediment organic matter content for the 80% shading absorption treatment, whereas, for 50% and the 20% treatments, the organic matter kept the same as the control group (Figure 3; Table 3).

**TABLE 3:** Generalized linear mixed models testing the effect of shading (0%, 20%, 50%, and 80%) on sediment organic matter content.

Organic matter content				
Parameters	Estimate	SE	*T*‐value	*p*‐Value
*Fixed effects*				
(Intercept)	−3.87	.16	−24.81	**<.001**
Shade 20%	0.07	.12	0.57	0.568
Shade 50%	0.08	.12	0.66	0.511
**Shade 80%**	**0.26**	**.12**	**2.10**	**.036**

Parameters		Variance	SD
*Random effects*			
ID	(Intercept)	0.017	0.129
Time	(Intercept)	0.011	0.105
Residual		0.038	0.194
Observations	136		
*N*_Time_	5		
*N*_ID_	28		

*Note*: The selected model structure was: shade + (1|time) + (1|id). The bold font indicates statistical significance (*p* < .05).

Abbreviations: ID, experimental structure identity; *N*, sample size; SD, standard deviation; SE, standard error.

### 3.3 Behavioral responses concerning the temperature

**FIGURE 4:**
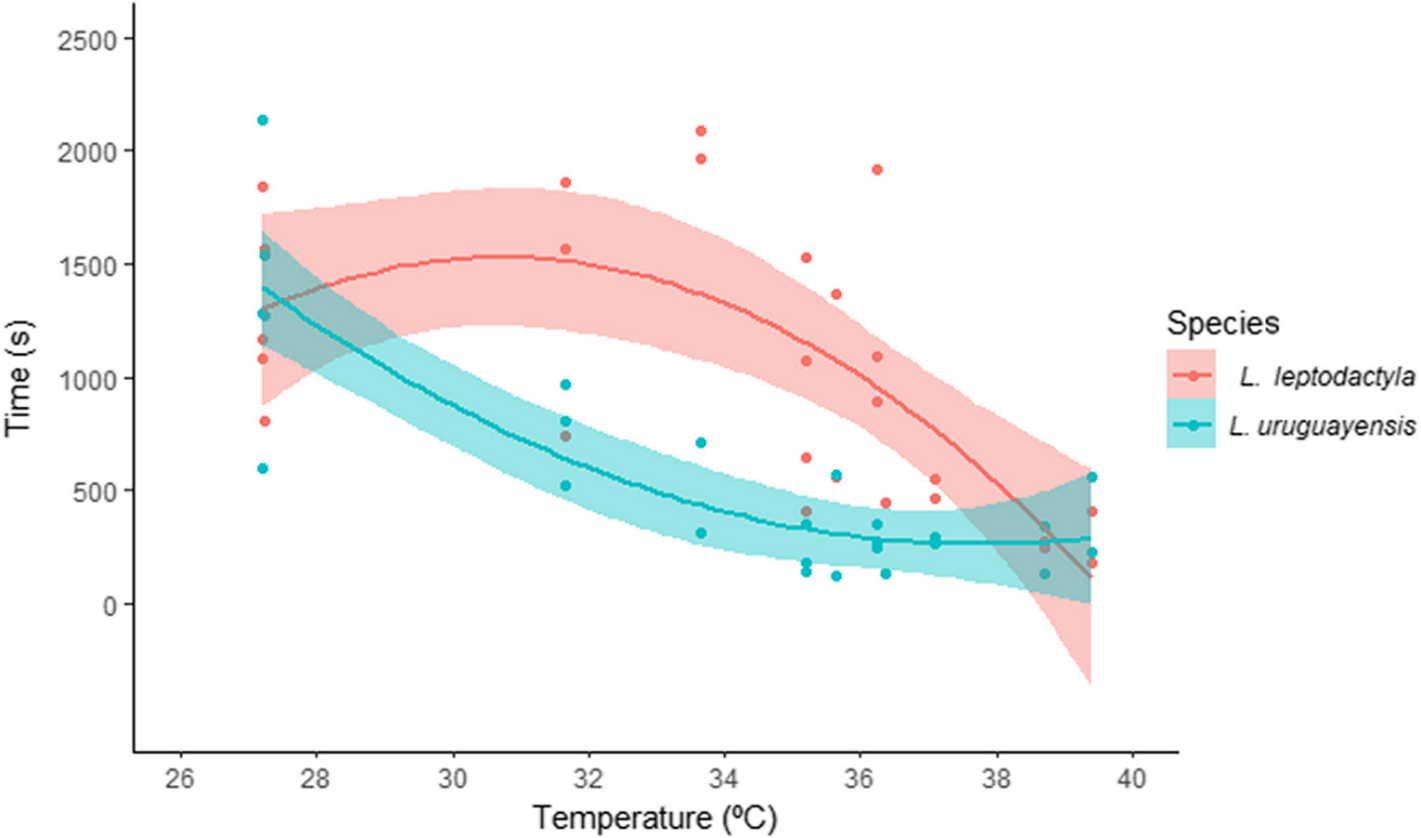
Time spent outside the burrow of males *Leptuca leptodactyla* and *Leptuca uruguayensis* concerning the sediment surface temperature. Dots are the time from each crab identity, while lines and shaded areas represent model polynomial (*x* ^2^) predictions ± SE.

Concerning the time crabs spent outside their burrows (see Appendix 2: Table A3 for model selection), there was an interaction between the fixed factor species and the covariate temperature. On the one hand, *L. leptodactyla* remained longer outside their burrows until a certain temperature (~31.1°C), reducing their activity in temperatures higher than that. On the other hand, the time *L. uruguayensis* spent outside the burrows followed an opposite pattern, with higher activities with cooler temperatures (Figure 4; Table 4).

**TABLE 4:** Generalized linear mixed models testing the effect of species and temperature on crab's time spent outside their burrow.

Time				
Parameters	Estimate	SE	*T*‐value	*p*‐Value
*Fixed effects*				
(Intercept)	6.76	.10	65.61	**<.001**
**Sp. (*L. uruguayensis*)**	**−0.73**	**.10**	**−7.24**	**<.001**
**Poly(Temperature, 2)1**	**−3.07**	**.74**	**−4.14**	**<.001**
**Poly(Temperature, 2)2**	**−2.68**	**.75**	**−3.57**	**<.001**
Sp. (*L. uruguayensis*)*Poly(Temperature, 2)1	**−1.46**	**.72**	**−2.01**	**<.001**
Sp. (*L. uruguayensis*)*Poly(Temperature, 2)2	**3.91**	**.71**	**5.46**	**<.001**

Parameters		Variance	SD
*Random effects*			
ID	(Intercept)	0.071	0.267
Residual		0.152	0.390
Observations	52		
*N*_ID_	26		

*Note*: The selected model structure was: specie + poly(temperature, 2) + specie:poly(temperarture, 2) + (1|id). The terms in bold indicate statistical significance (*p* < .05).

Abbreviations: ID, crab identity; *N*, sample size; SD, standard deviation; SE, standard error.

**FIGURE 5:**
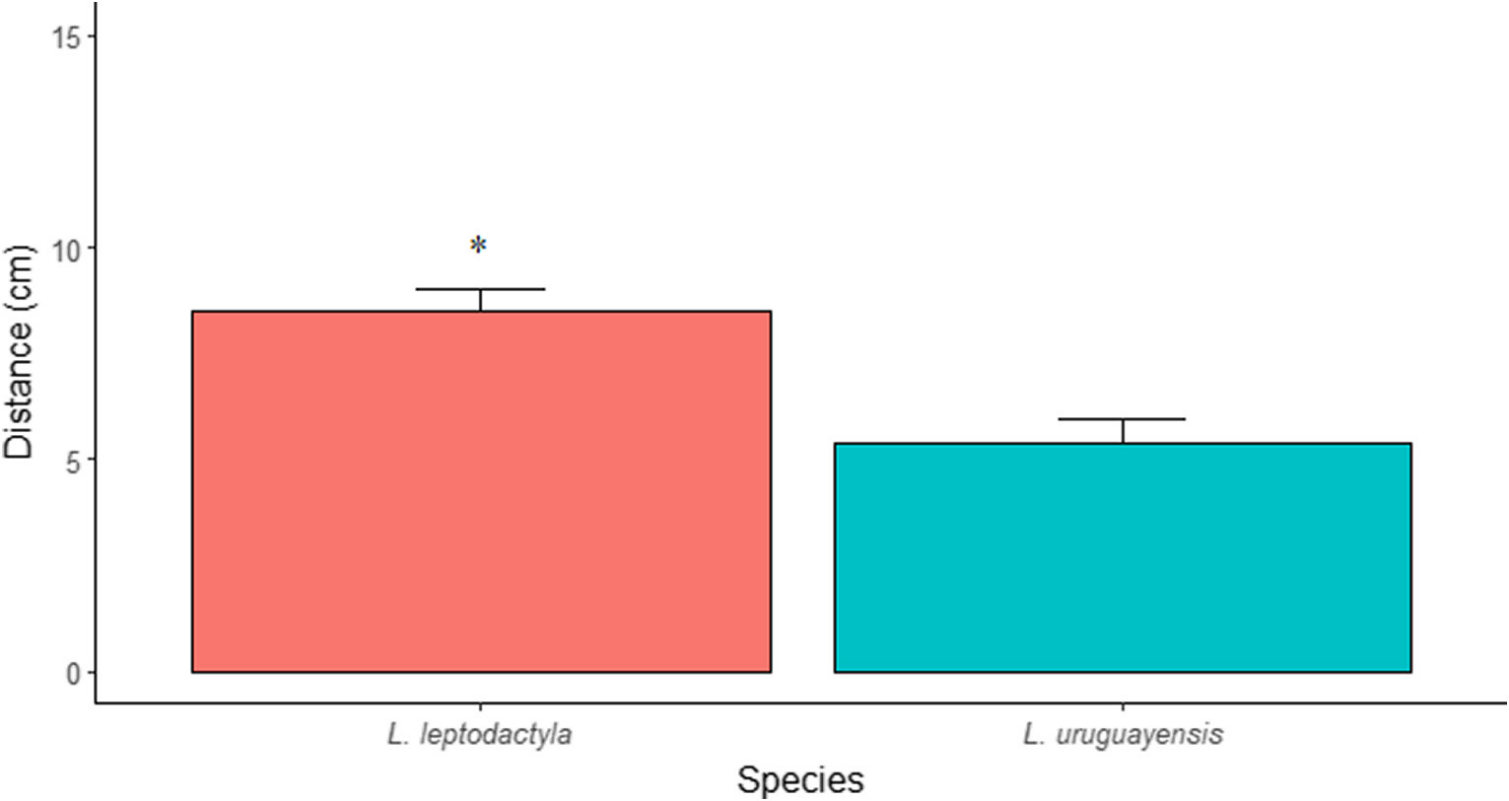
Mean ± SE of further traveled distance for *Leptuca leptodactyla* and *Leptuca uruguayensis*. The asterisk denotes statistical differences between species (*p* < .05).

For the crabs' traveled distance (see Appendix 2: Table A4 for model selection), there was neither an interaction of the fixed factors of temperature and species nor of temperature. The difference found was between species, as *L. leptodactyla* traveled further distances than *L. uruguayensis* (Figure 5; Table 5).

**TABLE 5:** Generalized linear mixed models testing the effect of species on crab's further distance traveled from their burrow entrance.

Distance				
Parameters	Estimate	SE	*T*‐value	*p*‐Value
*Fixed effects*				
(Intercept)	2.13	.08	24.82	**<.001**
**Sp. (*L. uruguayensis*)**	**−0.51**	**.09**	**−5.62**	**<.001**

Parameters		Variance	SD
*Random effects*			
ID	(Intercept)	0.05	0.21
Residual		0.13	0.36
Observations	52		
*N*_ID_	26		

*Note*: The selected model structure was: specie + (1|id). The bold font indicates statistical significance (*p* < .05).

Abbreviations: ID, crab identity; *N*, sample size; SD, standard deviation; SE, standard error.

### 3.4 Species physiological limitation

#### 3.4.1 Species water loss

**FIGURE 6:**
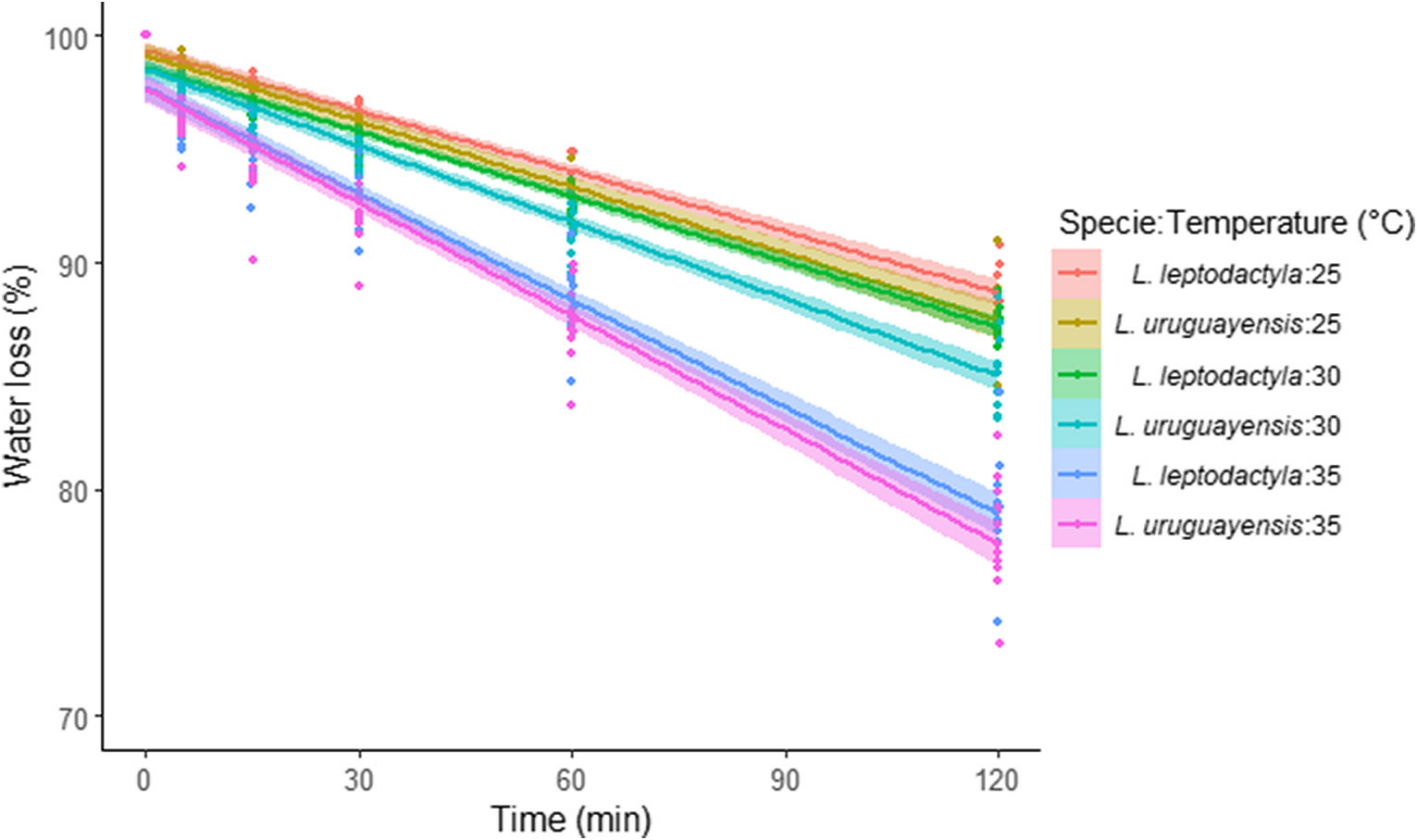
Water loss throughout time concerning the different temperatures of both species *Leptuca leptodactyla* and *Leptuca uruguayensis*. Dots are the water percentage from each crab identity, while lines and shaded areas represent model linear predictions ± SE.

Regarding species water loss throughout time according to the temperature (see Appendix 2: Table A5 for model selection), there was neither an interaction of any temperature and time nor species and time. There was an effect only of the temperature since crabs exposed to 30°C, and 35°C lost more water than the control group. In addition, irrespective of the temperature and species, crabs lost water over time (Figure 6; Table 6).

**TABLE 6:** Generalized linear mixed models testing the effect of temperature, specie, and time on crab's water loss.

Water loss				
Parameters	Estimate	SE	*Z*‐value	*p*‐Value
*Fixed effects*				
(Intercept)	3.64	.08	43.05	**<.001**
**Temperature 30**	**−0.20**	**.09**	**−2.14**	**.032**
**Temperature 35**	**−0.47**	**.09**	**−5.25**	**<.001**
Sp. (*L. uruguayensis*)	−0.05	.06	−0.78	.440
**Time**	**−0.01**	**.00**	**−14.06**	**<.001**
Temperature 30*Time	−0.00	.00	−0.23	.810
Temperature 35*Time	−0.00	.00	−1.65	.098
Sp. (*L. uruguayensis*)*Time	−0.00	.00	−0.68	.495

Parameters		SD	Corr
*Random effects*			
ID	(Intercept)	0.005	
	Time	0.0001	−.407
Observations	348		
*N*_ID_	58		

*Note*: The selected model structure was: temperature + specie + time + temperature:time + specie:time + (time|id). The bold font indicates statistical significance (*p* < .05).

Abbreviations: Corr, correlation coefficient; ID, crab identity; *N*, sample size; SD, standard deviation; SE, standard error.

For crabs' mortality at the end of the experiment, we observed no mortality at the end of the experiment to the 25°C of both species (0 out of 6), while *L. uruguayensis* had a higher mortality rate than *L. leptodactyla* to 30°C (10/11: 91%; and 2/11: 18%, respectively; *χ* ^2^ = 48.89, df = 1, *p* < .001), and 100% of mortality (12/12) to both species at 35°C.

#### 3.4.2 Thermal limits

The critical thermal limit of *L. leptodactyla* was different from *L. uruguayensis* (*t*‐test, *t*_28_ = 4.4876, *p* = .0001). *Leptuca leptodactyla* was more thermotolerant, reaching the loss of equilibrium at mean temperature ± SD of 43.11 ± 0.53°C while *L. uruguayensis* reached the loss of equilibrium at 42.28 ± 0.47°C.

## 4 DISCUSSION

In this study, it is shown that the coexistence between *L. leptodactyla* and *L. uruguayensis* is influenced by the diversification of microhabitat characteristics. This is because *L. leptodactyla* was more associated with nonshaded/warmer areas while *L. uruguayensis* to the shaded/cooler ones. To deal with thermal stress, the proportion of each species changed, indicating movement among microhabitats over the long‐term, as well as altering their behavior differently from each other in the short‐term. These effects are related to species‐specific physiological limitations. We conclude that biologically diverse ecosystems, such as intertidal regions from estuaries (Dame, 2008), might support the coexistence between closely related species by reducing niche overlap due to habitat heterogeneity.

The first step of our work was to confirm, by a manipulative approach, the long‐term effect of shading on species microhabitat selection. This confirmation is important because association tests point to a relationship between variables, but it is not possible to state that it is necessarily a cause‐effect relationship (De Grande et al., 2018; Underwood, 1997). Indeed, the shading gradient affected sediment surface temperature and luminous intensity as expected in all experimental groups, but temperatures barely exceeded 30°C in higher shading conditions during spring/early summer sunny days. In addition, the proportion of *L. leptodactyla* was reduced only for the 80% of shading level treatment, the one that also had lower temperatures and higher organic matter content in the sediment. Although our findings for both species are accordingly to the descriptive approaches that evaluate crabs' distribution considering shading as a binary factor (i.e., shaded or nonshaded; Checon & Costa, 2017, 2018; Thurman et al., 2013), we observed that the degree of shade is also important in this context, as there is a limit of shading that can alter the community (in this case from 80% of shading). Finally, despite the crabs' distribution being also related to the shade only when this affected sediment organic matter, it is known that *L. uruguayensis* did not show any preference for specific organic matter content (De Grande et al., 2018). Thus, the relationship between shade and temperature plays a relevant role in the fiddler crabs' distribution throughout the intertidal zone.

In the short‐term responses, we observed that *L. leptodactyla* stayed longer periods outside their burrows and traveled further distances than *L. uruguayensis* when both species were exposed to the sun. Nevertheless, this relationship is not linear. Instead, *L. uruguayensis* reduces its activities from temperatures above 27°C, while *L. leptodactyla* increase their activities above this temperature until reaching a limit, decreasing it in temperatures higher than 32°C. In any case, animals can adjust their behavior in unfavorable conditions to deal with thermal stress, reducing their activity on the sediment surface, as well as their opportunities for foraging and finding a mate. If conditions remain unfavorable for a longer period for some reason (e.g., the opening of clearings or vegetation growth) and behavioral adjustments do not pay off, both species might seek more thermally ideal microhabitats, as demonstrated in the shading experiment. Indeed, the temperature of the 80% shading treatment was approximately 27°C, a temperature that allows *L. uruguayensis* to stay active longer outside their burrows.

Similarly, the distribution of *A. mjoebergi* among microhabitats is influenced by a thermal window suitable for their activity on the sediment surface. This species inhabits a high intertidal zone where the mudflat are clearings surrounded by vegetation (Munguia et al., 2017). They tend to inhabit and to be active in nonshaded areas, but when the temperatures are too high (sediment temperature can easily trespass 40°C), their activity in shaded/cooler conditions is increased, without needing to retreat to their burrow as frequently as in nonshaded conditions, raising their chances to forage and to find a mate (Chou et al., 2019; Munguia et al., 2017). Consequently, partially‐shaded microhabitats are preferable for males of *A. mjoebergi* and higher crab densities can be found closer to vegetation borders (Chou et al., 2019; Munguia et al., 2017). Thus, although the role of thermal tolerance and associated microhabitats can influence fiddler crab opportunities, they can utilize habitat heterogeneity to find suitable places to maintain their optimum activities.

The distributional and behavioral patterns found herein are linked to species‐specific physiological limitations. Although the water loss of *L. uruguayensis* was similar to *L. leptodactyla*, irrespective of the temperature, the mortality of the first species was higher at 30°C. The CT_max_ experiment corroborates this difference in thermal tolerance, with the last species reaching the thermal limit with higher temperatures. Our findings are following what is predicted for these species. Higher temperatures cause *L. uruguayensis* to increase metabolic costs in comparison to *L. leptodactyla* (Vianna et al., 2020), as well as temperatures about 29°C affect the growth, reproduction, and survival of *L. uruguayensis* (De Grande et al., 2021). The thermal stress is also related to the species' latitudinal distribution pattern, since *L. uruguayensis* habits subtropical and temperate along the South American coast limited by higher temperatures in lower latitudes (De Grande et al., 2021), while *L. leptodactyla* habits neo‐tropical shores of the western Atlantic (Thurman et al., 2013), probably with the distribution limited by the lower temperatures in higher latitudes. Therefore, independently of sympatric interactions, physiological stressors are crucial in the spatial distribution of fiddler crabs (Nobbs & Blamires, 2017), not only on a latitudinal scale but also regarding differences in microhabitats.

In ecological communities, a necessary condition to support coexistence between species is ecological differentiation (Hardin, 1960). Habitat heterogeneity determines species abundance and richness (Erdős et al., 2018; Leviten & Kohn, 1980; Tews et al., 2004), and the coexistence of sympatric species is explained by habitat partition (Jorgensen, 2004; Liu et al., 2017; Palmer, 2003). Fiddler crabs are arguably a diverse patch‐specialist group with species inhabiting a great range of different microhabitats (Checon & Costa, 2017, 2018; Thurman et al., 2013; Weis & Weis, 2004). This favors the coexistence of so many species of fiddler crabs in the same ecosystem since it can be costly to live in mixed assemblies (Sanches et al., 2018). In summary, we show that the coexistence between estuarine intertidal closely related sympatric species is favored by the relationship between species' intrinsic factors, such as thermal stress tolerance, and the diversification of microhabitats, such as the shading degree. We conclude that the habitat heterogeneity from estuarine intertidal areas might favor species diversity in a general manner, due to reduced costs associated with habitat partition.

## AUTHOR CONTRIBUTIONS

**Fábio H. C. Sanches:** Conceptualization (lead); data curation (lead); formal analysis (lead); investigation (lead); methodology (lead); writing – original draft (lead). **Fernando R. De Grande:** Conceptualization (equal); formal analysis (equal); investigation (equal); methodology (equal); writing – review and editing (equal). **Tânia M. Costa:** Conceptualization (equal); funding acquisition (equal); methodology (equal); project administration (equal); supervision (equal); writing – review and editing (equal). **Rodrigo E. Barreto:** Conceptualization (equal); investigation (equal); methodology (equal); project administration (equal); supervision (equal); writing – review and editing (equal).

## CONFLICT OF INTEREST STATEMENT

There is no conflict of interest.

## Data Availability

All data are available in the figshare repository DOI: https://doi.org/10.6084/m9.figshare.22189918.

## APPENDIX 2 Generalized linear mixed models: model selection

### Shading effect on species microhabitat selection

**TABLE A1:** Comparison between the best 3 models from the *Shading effect on species microhabitat selection* experiment.

Model structure	AIC	ΔAIC	df	*w*_*i*_
Time + shade + time:shade + (time|id)	1449.5	0.0	11	0.7412
Time + shade + (time|id)	1451.6	2.1	8	0.2539
Time + shade + time:shade + (1|id)	1459.5	10.0	9	0.0049

*Note*: Models were ranked according to the lowest value from their Akaike information criterion (AIC) and higher Akaike weights (*w*_*i*_). ΔAIC is the difference between the AIC to the most parsimonious model, while df is the degree of freedom.

### Shading effect on sediment abiotic factors

**TABLE A2:** Comparison between the best 3 models for sediment organic matter content from the *Shading effect on sediment abiotic factor* experiment.

Model structure	AIC	ΔAIC	df	*w*_*i*_
Shade + (1|time) + (1|id)	−1065.7	0.0	7	1
Shade + (1|time)	−1009.9	55.8	6	<0.001
Shade + time + (1|id)	−982.5	83.2	7	<0.001

*Note*: Models were ranked according to the lowest value from their Akaike information criterion (AIC) and higher Akaike weights (*w*_*i*_). ΔAIC is the difference between the AIC to the most parsimonious model, while df is the degree of freedom.

### Behavioral responses concerning the temperature

**TABLE A3:** Comparison between the best 3 models for the time crabs spent outside their burrows of the *Behavioral responses concerning the temperature* experiment.

Model structure	AIC	ΔAIC	df	*w*_*i*_
Specie + temperature + specie:temperature + (1|id)	757.9	0.0	6	0.45
Specie + temperature + (1|id)	758.0	0.1	5	0.43
Specie + temperature + specie:temperature + (temperature|id)	760.6	2.7	8	0.12

*Note*: Models were ranked according to the lowest value from their Akaike information criterion (AIC) and higher Akaike weights (*w*_*i*_). ΔAIC is the difference between the AIC to the most parsimonious model, while df is the degree of freedom.

**TABLE A4:** Comparison between the best 3 models for the crabs' traveled distance of the *Behavioral responses concerning the temperature* experiment.

Model structure	AIC	ΔAIC	df	*w*_*i*_
Specie + poly(temperature, 2) + specie:poly(temperature, 2) + (1|id)	739.0	0.0	8	1
Specie + temperature + specie:temperature + (1|id)	757.9	19.0	6	<0.001
Specie + temperature + (1|id)	758.0	19.1	5	<0.001

*Note*: Models were ranked according to the lowest value from their Akaike information criterion (AIC) and higher Akaike weights (*w*_*i*_). ΔAIC is the difference between the AIC to the most parsimonious model, while df is the degree of freedom.

### Species physiological limitation

**TABLE A5:** Comparison between the best 3 models for the *species water loss* experiment.

Model structure	AIC	ΔAIC	df	*w*_*i*_
Temperature + specie + time + temperature:time + specie:time + (time|id)	−1688.5	0.0	12	0.71
Temperature + specie + time + temperature:time + (time|id)	−1685.1	3.4	11	0.13
Temperature + specie + time + specie:time + (time|id)	−1684.1	4.4	10	0.08

*Note*: Models were ranked according to the lowest value from their Akaike information criterion (AIC) and higher Akaike weights (*w*_*i*_). ΔAIC is the difference between the AIC to the most parsimonious model, while df is the degree of freedom.
